# Calculation of alpha particle single-event spectra using a neural network

**DOI:** 10.3389/fonc.2024.1394671

**Published:** 2024-10-02

**Authors:** Layth Alkhani, Jason P. Luce, Pablo Mínguez Gabiña, John C. Roeske

**Affiliations:** ^1^ Department of Bioengineering, Stanford University, Stanford, CA, United States; ^2^ Department of Radiation Oncology, Stritch School of Medicine, Loyola University Chicago, Maywood, IL, United States; ^3^ Department of Medical Physics and Radiation Protection, Gurutzeta/Cruces University Hospital, Biocruces Health Research Institute, Barakaldo, Spain

**Keywords:** microdosimetry, alpha particles, neural networks, radiation, machine learning

## Abstract

**Introduction:**

A neural network was trained to accurately predict the entire single-event specific energy spectra for use in alpha-particle microdosimetry calculations.

**Methods:**

The network consisted of 4 inputs and 21 outputs and was trained on data calculated using Monte Carlo simulation where input parameters originated both from previously published data as well as randomly generated parameters that fell within a target range. The 4 inputs consisted of the source-target configuration (consisting of both cells in suspension and in tissue-like geometries), alpha particle energy (3.97–8.78 MeV), nuclei radius (2–10 μm), and cell radius (2.5–20 μm). The 21 output values consisted of the maximum specific energy (z_max_), and 20 values of the single-event spectra, which were expressed as fractional values of z_max_. The neural network consisted of two hidden layers with 10 and 26 nodes, respectively, with the loss function characterized as the mean square error (MSE) between the actual and predicted values for z_max_ and the spectral outputs.

**Results:**

For the final network, the root mean square error (RMSE) values of z_max_ for training, validation and testing were 1.57 x10^-2^, 1.51 x 10^-2^ and 1.35 x 10^-2^, respectively. Similarly, the RMSE values of the spectral outputs were 0.201, 0.175 and 0.199, respectively. The correlation coefficient, R_2_, was > 0.98 between actual and predicted values from the neural network.

**Discussion:**

In summary, the network was able to accurately reproduce alpha-particle single-event spectra for a wide range of source-target geometries.

## 1 Introduction

Targeted radionuclide therapy (TRT) first saw its application over 80 years ago and since then it has grown to become an area of great interest as advances in cancer biology, bioengineering, and radiochemistry have dramatically increased the potential of this modality (1). In contrast to other therapeutic techniques, TRT is based on the use of high-affinity molecules as carriers of radionuclides to tumor cells (2–5). The nature of this technology thus creates an approach for tumor therapy that is personalized to each individual patient, making strides towards a future that mitigates risks for normal tissue damage by delivering a highly conformal absorbed dose to the tumor (1). There are three classes of radionuclides that have been considered for TRT: alpha emitters, beta emitters, and Auger electron emitters (1, 6–8). Of these, alpha emitters have inherent properties that make them favorable in comparison to the others, pushing some to regard them as a potential “magic bullet” (5). The advantages of alpha emitters include: 1) high energy (3–9 MeV); 2) a densely ionizing track; 3) short path length (40–90 µm, corresponding to 2–10 cell diameters), 4) high linear energy transfer (LET) and 5) independence of dose rate and oxygen effects (6, 8). The practical implications of these characteristics allow for alpha particles to sterilize individual tumor cells solely from self-irradiation which is not possible with the more widely used beta-particle emitters (6, 8). Moreover, alpha-particle emitters have shown promising clinical results, for example in the treatment of metastatic castration-resistant prostate cancer (mCRPC) (9, 10).

Despite their efficacy, the stochastic nature of alpha emitters which deposit energy in small, subcellular targets causes alpha particle dosimetry to be particularly challenging (6, 8). Dosimetry – i.e. the measurement of the amount of energy deposited per unit mass - has been in regular use in conventional radiotherapy (11). However, the minute scale at which radionuclides operate introduces significant challenges (variability in energy deposition) (11). At the cellular level, the amount of energy deposited in the critical target (i.e., cell nucleus) depends on the combination of both the cell geometry and the alpha particle’s path through that target (6, 8). Additionally, some cell nuclei may receive multiple alpha particle hits while others receive few or none. Consequently, the stochastic nature of alpha particles hits combined with the non-uniform energy deposition may result in a local deviations exceeding 20% in the energy deposited within cell nuclei (6, 8, 12). This variation was established through Monte Carlo simulations and analytical calculations which showed that such variations are common due to the random nature of alpha particle interactions at the microscopic level necessitating the use of microdosimetry to accurately measure the energy deposited within cellular targets (6, 8, 12).

Microdosimetry considers the stochastic nature of the energy deposited per unit mass. Specific energy (z) is the microdosimetric analog of absorbed dose (D) and is given by:


(1) $$z=\;\frac{\text{ϵ}}{m}$$

where ϵ is the energy deposited within the cellular target and *m* is the mass of that target. Similar to absorbed dose, specific energy has units of Gy. A fundamental quantity in microdosimetry is the single-event specific-energy spectrum (f_1_(z_1_)) which is the frequency distribution of specific energy deposited within the target for exactly one alpha particle hit (13). The multi-hit spectrum for n alpha particle hits can be determined by performing multiple convolutions of the single-event spectrum (14). Of significance, single-event spectra can be used to estimate cell survival using:


(2) $$S\left(D\right)=\;e^{-\langle n\rangle \left[1-T_{1}\left(z_{o}\right)\right]}$$

where S(D) is the fraction of cells that survive after receiving an average dose D, <n> is the average number of hits to the cellular target, T_1_(z_o_) is the Laplace transform of the single-event spectrum, f_1_(z_1_), and z_o_ is the specific energy deposited in a cell to reduce survival to 1/e (15, 16).

Alternatively, the first and second moments of the single-event spectrum can also be used to estimate cell survival (15, 17–19). The first moment of the single-event spectrum given by:


(3) $$\langle z_{1}\rangle \;=\;\int_{z=0}^{\infty }z_{1}\;f_{1}\left(z_{1}\right)dz_{1}$$

while the second moment is defined as:


(4) $$\langle z_{1}^{2}\rangle \;=\;\int_{z=0}^{\infty }z_{1}^{2}\;f_{1}\left(z_{1}\right)dz_{1\;.}$$

Using these moments, T_1_(z_o_) from Equation 2 can approximated by (20):


(5) $$T_{1}\left(z_{o}\right)\approx \text{exp }\left(-\frac{\langle z_{1}\rangle }{z_{o}}-\;\frac{\langle z_{1}^{2}\rangle \;-\;{\langle z_{1}\rangle }^{2}}{2z_{o}^{2}}\right)$$

Relating to classical microdosimetry, <z_1_> is the frequency mean (z_F_) while <z_1_^2^>/<z_1_> is the dose mean (z_D_) specific energy per event described by Kellerer (12) and Roesch (21). Moreover, these quantities have been related to cellular damage from alpha particle emitters (20, 22).

In order to calculate microdosimetric spectra, an analytical or Monte Carlo (MC) approach can be used (6, 8). The complexity that arises from many alpha-particle emissions combined with the greater flexibility which is provided by the MC simulation makes this approach favorable (8, 23). A recent study conducted by our group determined that a neural network (NN) could be trained to accurately calculate <z_1_> and <z_1_^2^> for alpha-particle microdosimetry calculations (24). The goal of our study is to take the next step forward and create a NN that can accurately and efficiently calculate the entire single-event specific-energy spectra. In this paper we present a novel approach where machine learning methods are utilized to “teach” a network to produce microdosimetric spectra from a set of input parameters (nucleus/cell size, initial alpha particle energy, and source/target geometry). This approach, the first of its kind, would allow for the network, once trained, to quickly and easily produce microdosimetric spectra for configurations that the network was not trained on, particularly, novel radionuclides (different initial alpha particle energies) and different combinations of cell/nuclear dimensions.

## 2 Methods

### 2.1 Data set

**Figure 1:**
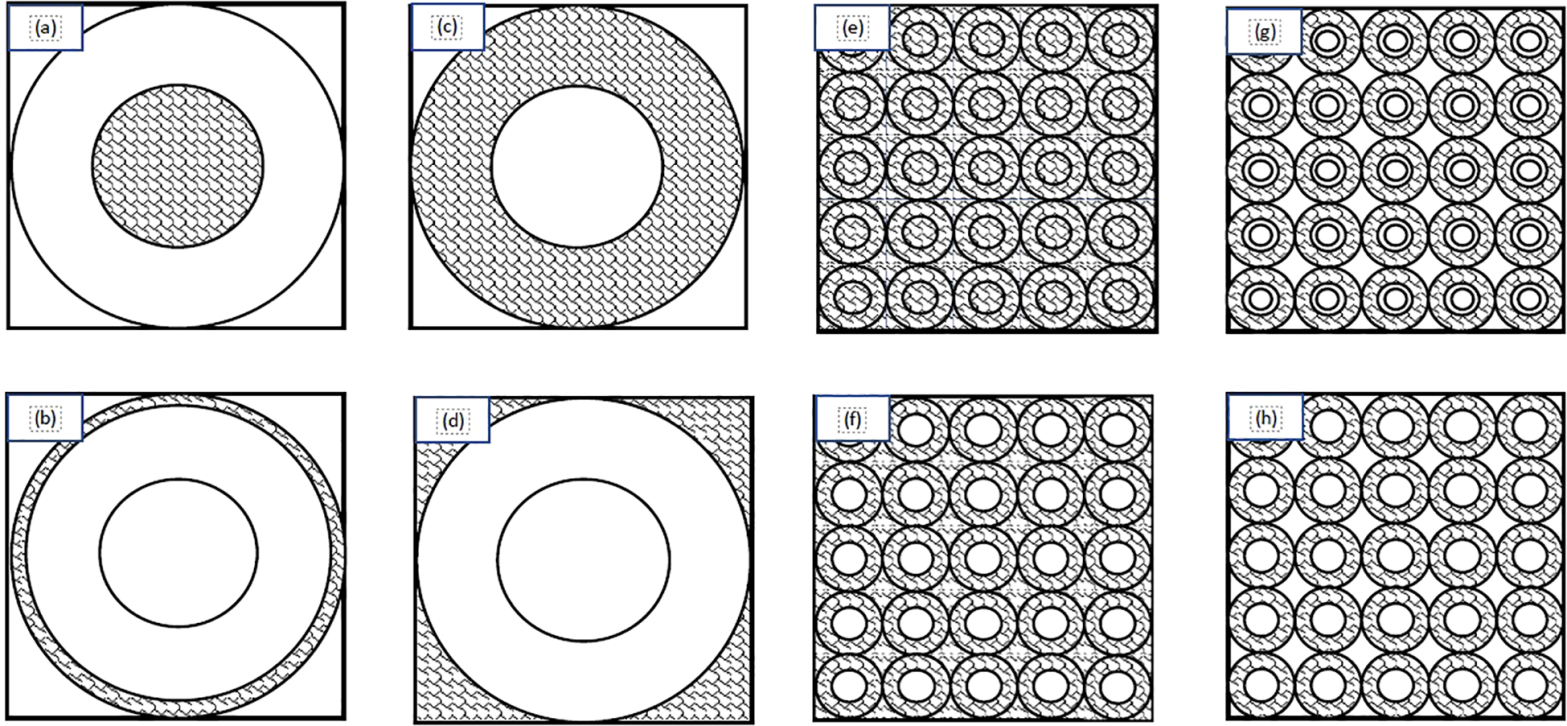
Schematic diagram of the source/target distributions considered in this study: **(A)** source within the cell nucleus; **(B)** source on the cell surface; **(C)** source in the cell cytoplasm; **(D)** source outside of the cell; **(E)** uniform everywhere; **(F)** uniform everywhere except the nuclei; **(G)** in a spherical shell between the cell membrane and 1.25 times the cell nucleus radius; **(H)** only in the cytoplasm. Adapted from Wagstaff et al. (24).

In order to train the NN, the first step was to create an adequate training data set. This study collected data for the training/validation/testing from two data sets. The initial data set was produced using input values from a previously published paper (20). This paper was originally produced to provide the first and second moments of the single-event specific-energy spectrum of common alpha particle emitters to provide basic microdosimetry information. For the purposes of our study Tables I-IV were used to generate the first data set (20). These tables were included because the tabulated data cover the range of cell and nuclear radii as well as energies that are most likely to be encountered in TRT. Specifically, the nuclear radii ranged from 2-10 μm - including 2, 3, 4, 5, 6, 8, and 10 μm - and cell radii from 3-15 μm (25). Cells and nuclei were considered spherical and concentric. The source/target geometries considered in this study included: activity confined to the cell nucleus, activity in the cytoplasm, activity on the cell surface, and a uniform activity outside the cell (**Figures 1A–D**). The alpha particle energies from these tables corresponded to those of radionuclides considered to be suitable for therapeutic applications: terbium-149 (3.97 MeV), polonium-210 (5.3 MeV), astatine-211 (5.867 MeV), bismuth-212 (6.05 MeV), polonium-211 (6.73 and 7.45 MeV), polonium-213 (8.37 MeV), and polonium-212 (8.78 MeV) (26). Other radionuclides considered for alpha particle therapy, such as thorium-227, actinium-225, radium-223 and bismuth-213, have energies within this range (26). The strength of this data set is the range of values which cover most clinical applications and the grid-like structure of the inputs. One drawback, however, is that a grid-like structure’s accuracy can be severely reduced when dealing with edge cases or an input which is distant from all trained values. Use of these data resulted in 160 combinations of alpha particle emission energies, source/target geometries, cellular and nuclear radii. To account for the limitations of the previous data set another 160 values were calculated which utilized energy, nucleus radii, and cell radii that fell within the previously outlined values (energy 3.97-8.78 MeV; nuclear radii 2-10 μm; cell radii 3-15 μm) and were generated using a random number generator. The Monte Carlo (MC) algorithm used to produce these spectra is described in the **Appendix** (27).

In order to account for more realistic/complex geometries such as clusters of cells (**Figures 1E–H**) microdosimetric spectra were produced using a code described previously (See **Appendix**) (19). These geometries simulated layers of tissue wherein for each case there is a central spherical nucleus target. The central target is surrounded by a packed grid of cells forming a plane that are stacked on top of one another to form layers simulating tissue. The sources consisted of a uniform distribution of activity, activity everywhere except the cell nuclei, activity located in a spherical shell between the cell membrane and 1.25 times the cell nucleus radius, and activity only in the cytoplasm. The energies used match the energies from the previous data ranging from 3.97 - 8.78 MeV with exceptions being the addition of 6.4 and 7.l1 MeV as well as substitutions of 5.8 and 8.4 for 5.867 and 8.37 MeV respectively. Nucleus radii ranged from 2-10 μm and cell radii ranged from 2.5-20 μm with cell-to-nucleus ratios of 1.25, 1.5, 1.75, and 2. The additional data set broadens the network by including more energies, cell and nucleus sizes, as well as the more realistic tissue configurations.

Together, the data sets combined from both methods consisted of 2264 unique combinations of the source-target configuration, energy, cell and nuclear radii. These input values were run through MC simulations that are described in the **Appendix**. The result of each simulation was a single-event specific-energy spectrum with 20 output values (representing the bins of the energy spectrum histogram). Each spectrum was normalized such that the area under the curve was unity. These spectra were used to train the network as described in the next section. An additional value, z_max_, which is defined as the maximum specific energy for each spectrum was also recorded. This value allows one to determine the scale for the x-axis of the spectrum. That is, for the k^th^ value of the spectrum, the corresponding z value is given by kz_max_/20.

### 2.2 Neural network structure and training

The development of the NN used for this study was done using the MATLAB^®^ Deep Learning Toolbox™ (The Mathworks Inc., Natick, MA, USA). The process consisted of creating, testing, and adjusting a NN until it converged to produce the best results. For the purposes of this study, the number of hidden layers and number of nodes within each layer were varied to produce the optimal NN.

The Levenberg-Marquardt algorithm was used for training which combines both the gradient descent method and the Gauss-Newton method to solve nonlinear least squares curve-fitting problems (28). This algorithm was used as it is the most efficient in comparison to other techniques for networks of our size (29). The associated error function for this algorithm is the mean squared error (MSE) where a value close to zero is favorable. This is optimal for our case as using measurements which automatically re-scale and normalize error contributions are favorable for cases where multiple outputs have different scales (30).

To improve network accuracy, the data set had to undergo several transformations prior to training:

1. The highly skewed nature of the specific energy spectra warranted the use of the natural logarithm (Ln) transformation which was performed on the entire dataset (31). Additionally, a value of unity was added to all values prior to the Ln transformation to account for several target outputs with a value of 0. This transformation created an approximately normal distribution in the data, significantly improving NN performance.
2. A scaling factor of 4, determined by the ratio of z_max_:spectral values, was separately applied to the z_max_ value of the data set. Of the 21 network outputs, 20 corresponded to y-values of the spectrum while one output, z_max_, represented the maximum value for the x-axis. In general, the magnitudes of z_max_ values were significantly smaller than the other outputs, warranting a scaling factor being applied to ensure the network weights were appropriately updated from errors in z_max_ predictions.

**Figure 2:**
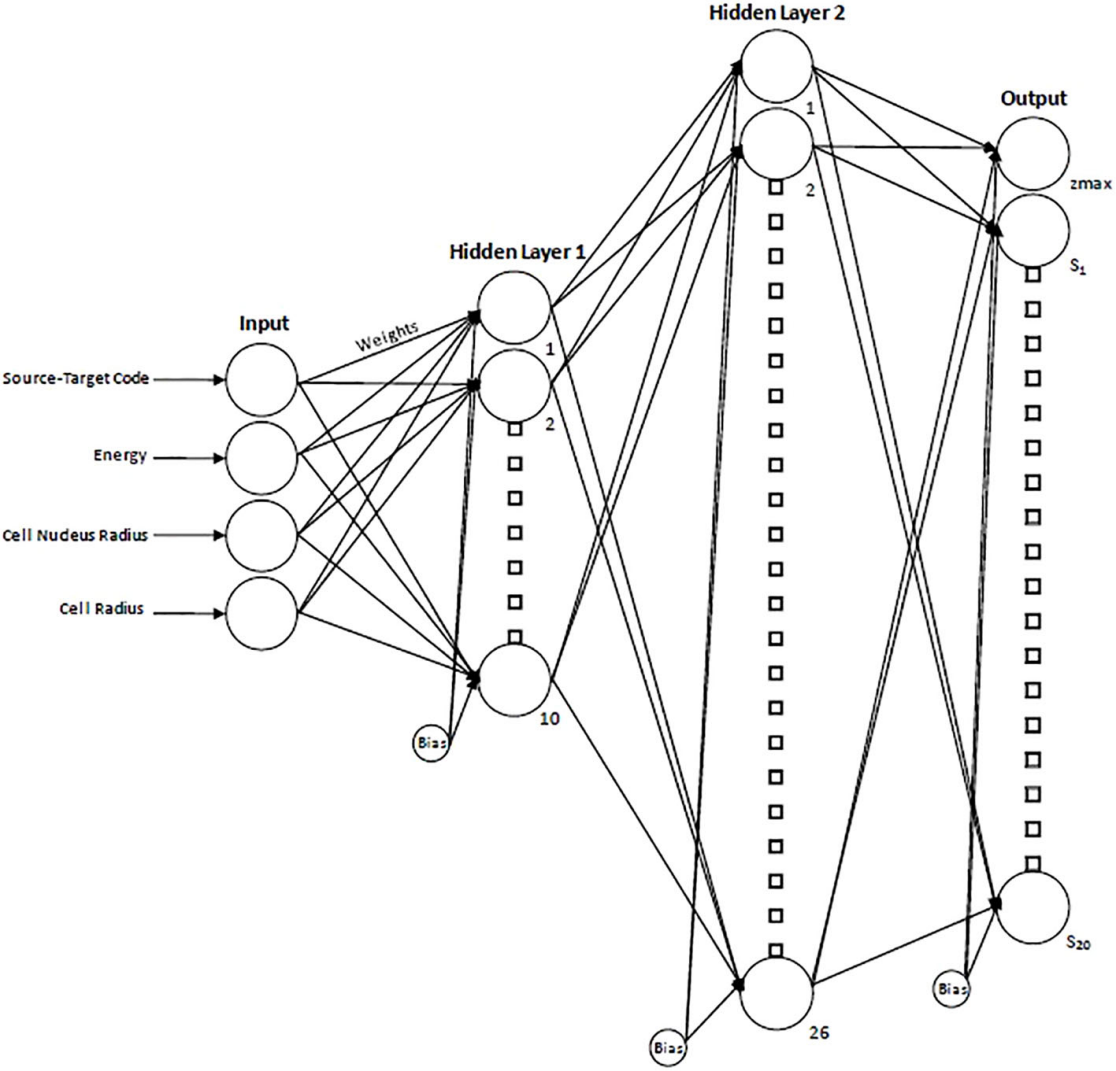
Schematic diagram of the resultant neural network displaying the 4 input parameters, hidden layers, and resultant output. For the output layer, z_max_ indicates the maximum specific energy of the single-event spectrum and provides a scaling factor for the x-axis, while S_1_…S_20_ indicate the 20 spectral values.

To determine the optimal number of hidden layers/node combinations, an initial empirical approach was employed where, through iterative testing and performance evaluation, it was determined that single-digit-sized layers were suboptimal and resulted in poorer performance. From there, a loop was created to iterate over all possible combinations of nodes in networks containing two hidden layers (double-digit layer sizes). The number of nodes in the first hidden layer ranged from 10-20 to while the number of nodes in the second hidden layer ranged from 10-40. The network was trained for each combination of nodes in the first and second hidden layers, and the root mean squared error (RMSE) was recorded. After this was completed, a minimum was found based on the calculated values. The optimal NN size was determined to be two layers with 10 nodes in the first layer and 26 in the second layer as shown schematically in **Figure 2**. The final network consisted of a total of 876 adjustable weights and biases. The spectral data were partitioned with 70% of the data used for training, 15% used for validation and 15% used for testing (32).

## 3 Results

**Figure 3:**
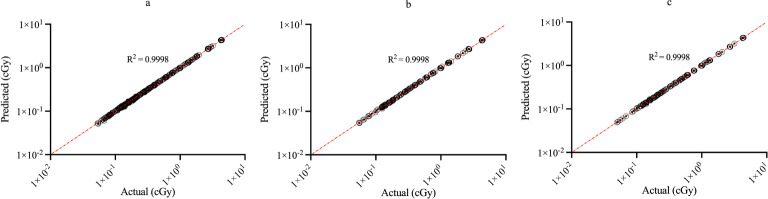
Regression plots demonstrating the degree of agreement between predicted values and known values for z_max_ for **(A)** training; **(B)** validation and **(C)** testing data. Each point represents actual value (x-axis) and the value predicted by the network (y-axis).

**Figure 4:**
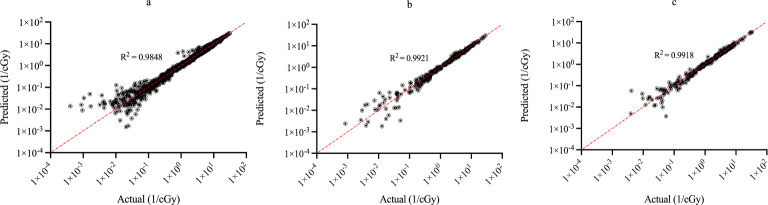
Regression plots demonstrating the degree of agreement between predicted values and known values for the single-event spectra values for **(A)** training; **(B)** validation and **(C)** testing data. Each point represents actual value (x-axis) and the value predicted by the network (y-axis).

**Figure 5:**
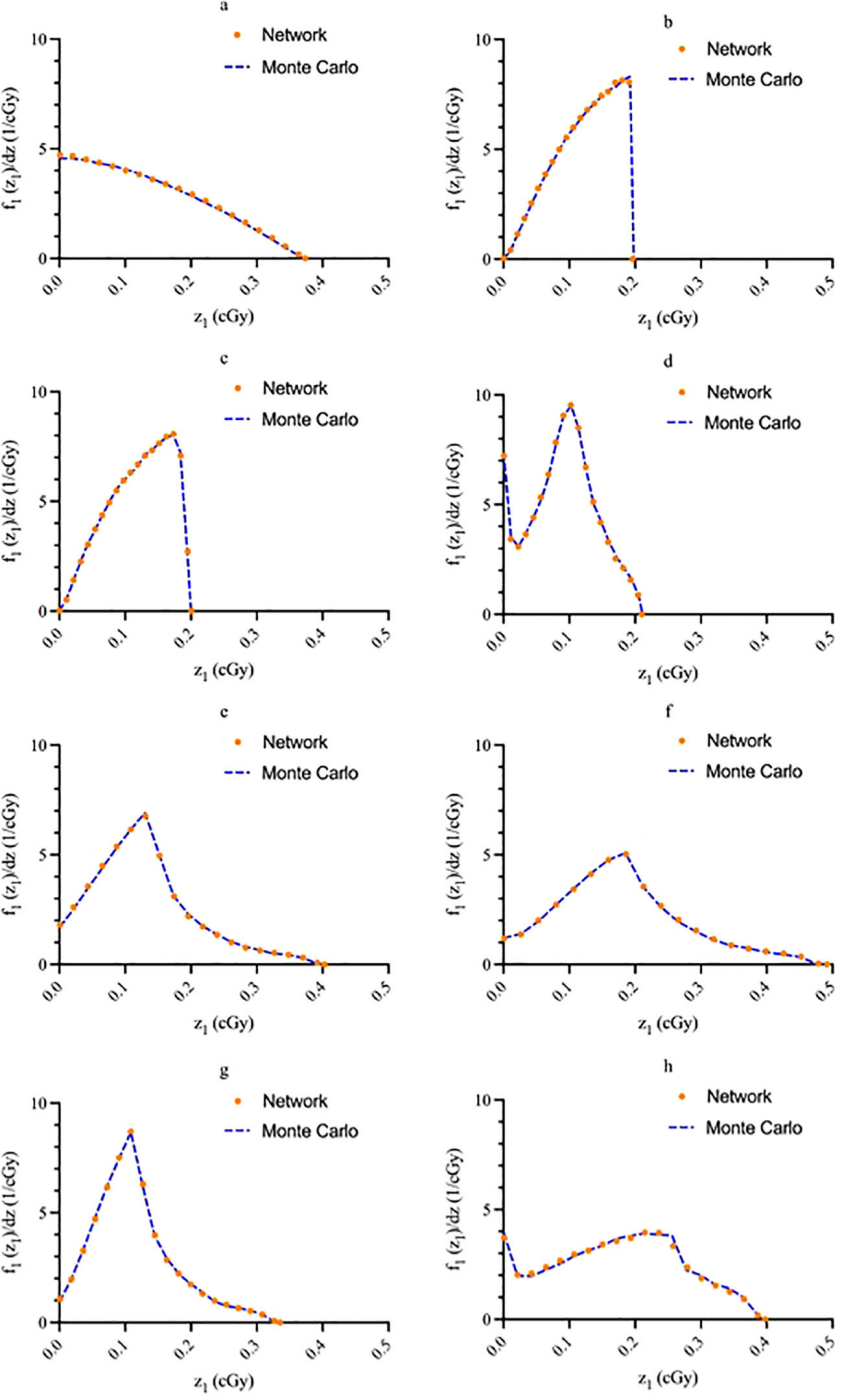
Sample network output graphs illustrating agreement between predicted values and known values for **(A)** source within the cell nucleus; **(B)** source on the cell surface; **(C)** source in the cell cytoplasm; **(D)** source outside of the cell; **(E)** uniform everywhere; **(F)** uniform everywhere except the nuclei; **(G)** in a spherical shell between the cell membrane and 1.25 times the cell nucleus radius; **(H)** only in the cytoplasm. Graph **(A)** energy: 3.97 MeV, nucleus size: 5 μm, cell size: 10 μm; **(B)** energy: 4.34 MeV, nucleus size: 7.02 μm, cell size: 11.06 μm; **(C)** energy: 5.867 MeV, nucleus size: 6 μm, cell size: 12 μm; d) energy: 6.05 MeV, nucleus size: 8 μm, cell size: 15 μm; **(E)** energy: 8.4 MeV, nucleus size: 6 μm, cell size: 7.5 μm; **(F)** energy: 6.9 MeV, nucleus size: 5.5 μm, cell size: 11 μm; **(G)** energy: 8.6 MeV, nucleus size: 6.5 μm, cell size: 13 μm; **(H)** energy: 3.97 MeV, nucleus size: 6 μm, cell size: 9 μm.

**Figure 3** shows the regression plots corresponding to the training, validation, and test data for the z_max_ value while **Figure 4** shows the resultant graphs for the 20 spectral outputs. For each plot, the x-axis corresponds to the ground truth values which were calculated using MC simulations and the y-axis corresponds to the NN output. The best-fit line is shown along with the R^2^ value for each plot. In general, the slope of the best-fit line is close to unity with only small y-offset values. Combined with R^2^ > 0.98 for all plots indicates good agreement between actual and predicted values. The RMSE values for z_max_ were 1.57 x10^-2^, 1.51 x 10^-2^ and 1.35 x 10^-2^ for training, validation and testing data, respectively. Similarly, the RMSE values for the spectral output were 0.201, 0.175 and 0.199, respectively. Of note, the z_max_ values (**Figure 3**) show good agreement across the entire range which is important since this value is used to provide accurate scaling of the x-axis for the resultant spectra. **Figure 4** shows some deviations for smaller spectral values (< 0.1). However, differences on this scale do not significantly alter the spectra nor impact the area-under-the-curve of these spectra. As shown in **Figure 5**, these spectra typically have values ranging from 1-10 cGy^-1^. Hence small deviations on the order of 0.1 or less do not have a large impact on the spectrum, nor in calculating the area-under-the-curve.

The single-event specific-energy spectra generated by the network are compared with MC data for a random sample of test data in **Figure 5**. All graphs have an area under the curve of unity. The solid blue line represents the MC data while the orange dots superimposed on the line represent the NN outputs. In general, good agreement between the NN and MC data is noted for all source-target geometries.

**Figure 6:**
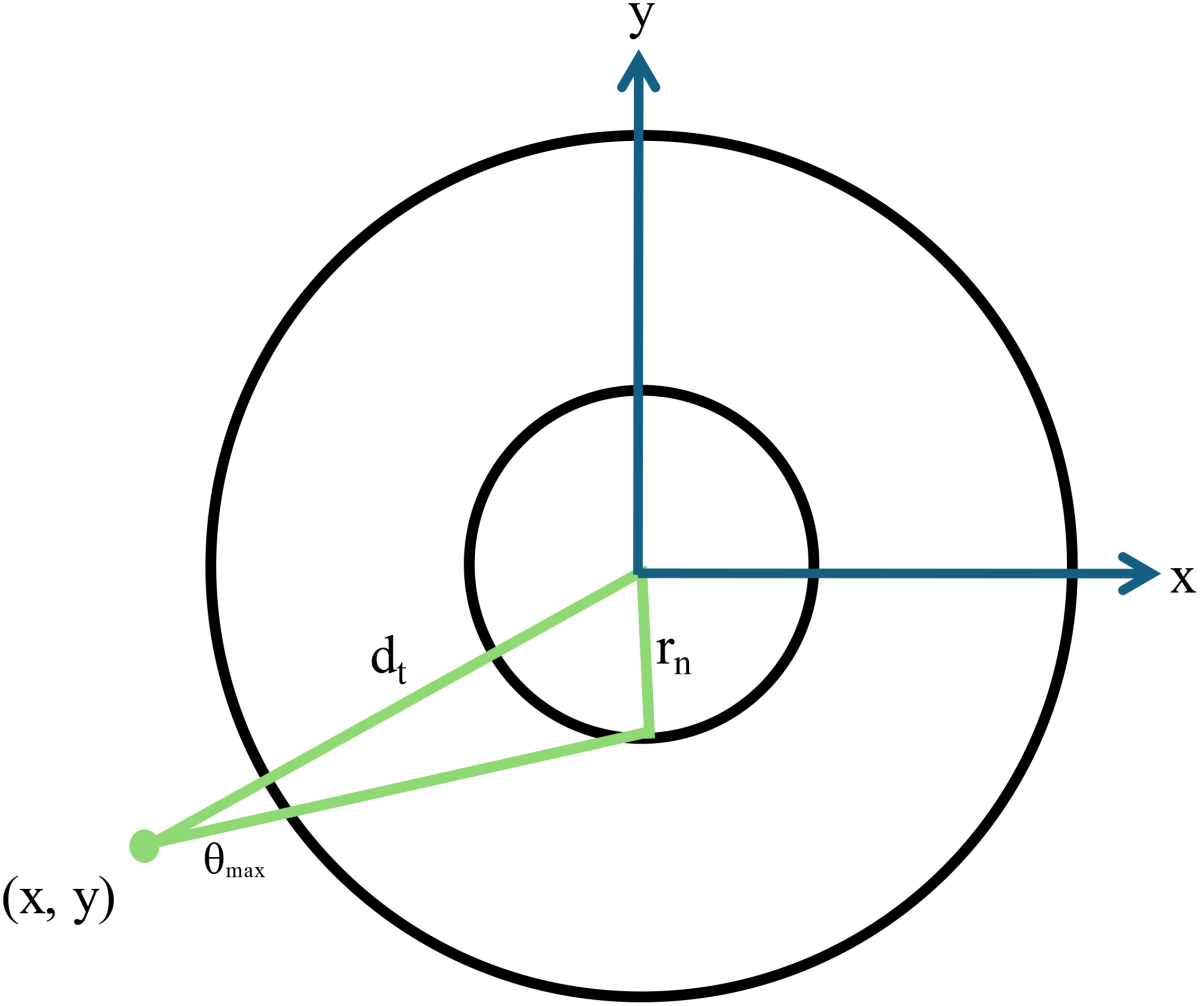
A schematic diagram showing a two-dimensional representation of the cell geometry used for Monte Carlo calculations. The cell nucleus has a radius r_n_ and the maximum angle that an alpha particle at point x,y subtends with the cell nucleus is given by θmax. The distance from the point of emission to the center of the nucleus is given by d_t_.

**Figure 7:**
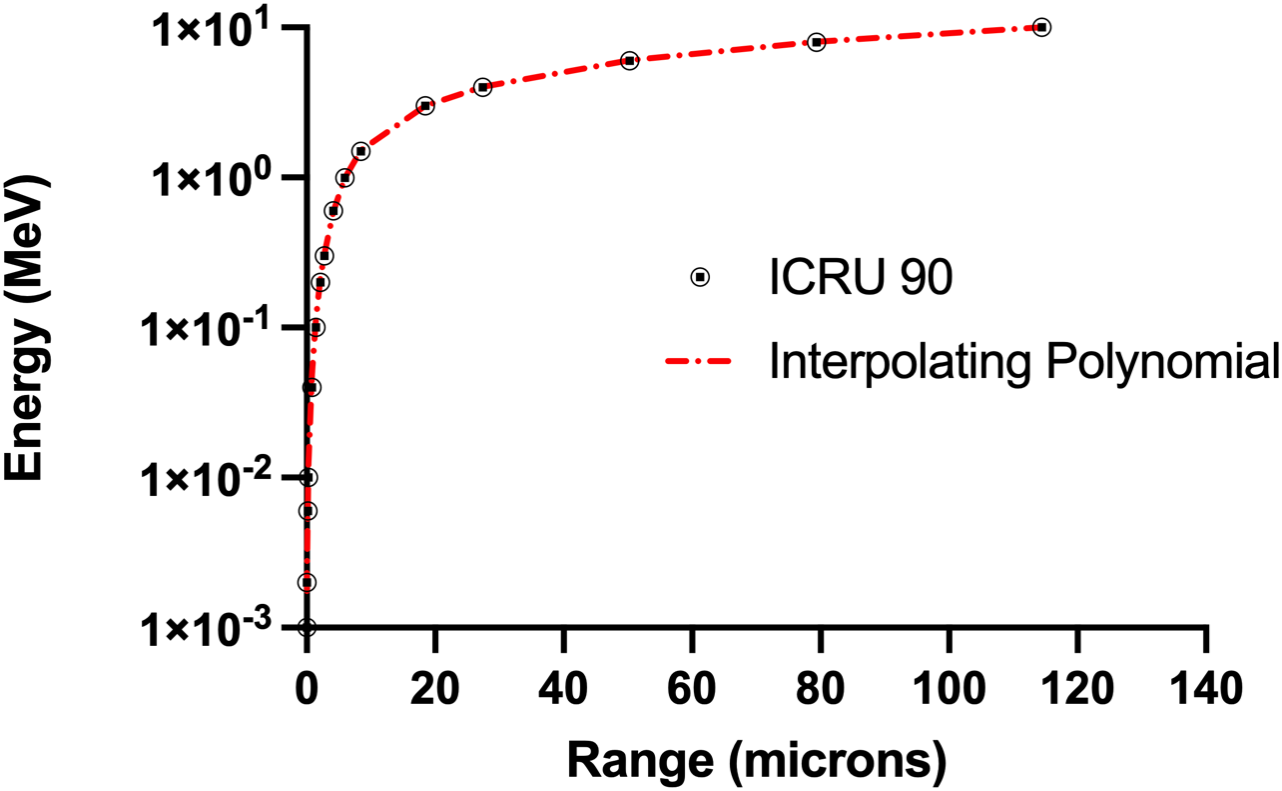
Comparison on ICRU 90 range-energy relationship for alpha particles with the curve fit based on interpolating polynomials in Equations A.2 and A.3 (R^2^ = 0.999).

**Table 1** shows the error distribution for tabulated <z_1_> and <z_1_^2^> using Equations 3, 4 based on the spectra generated using the NN vs. those generated from MC spectra. The mean for all three partitions (training, validation, and test) was centered near 0 with the <z_1_^2^> mean % error being slightly larger than the <z_1_> mean % error. The mean percent error for the training set was 0.14% for <z_1_> and 0.20% for <z_1_^2^>. For testing, the average <z_1_> error was 0.08% and <z_1_^2^> was 0.09%. The standard deviation (SD) and range of values also followed similar trends with <z_1_^2^> values being higher in all cases. The center of the values allowed for an approximately normal distribution and as a result a 95% confidence interval was calculated.

**Table 1:** Statistics summarizing the percent difference between <z_1_> and <z_1_^2^> calculated from single-event spectra predicted by the network vs. published Monte Carlo simulation values. SD = standard deviation.

	Training		Validation		Testing	
	<z_1_>	<z_1_^2^>	<z_1_>	<z_1_^2^>	<z_1_>	<z_1_^2^>
Mean % Error	0.14%	0.20%	-0.05%	-0.06%	0.08%	0.09%
% Error SD	2.77%	4.17%	2.82%	4.25%	2.46%	3.69%
95% Confidence Interval	[-4.29%, 5.99%]	[-6.48%, 8.82%]	[-5.02%, 4.83%]	[-7.24%, 7.49%]	[-4.71%, 5.15%]	[-6.67%, 7.93%]

## 4 Discussion

In this study we developed a NN capable of producing single-event specific energy spectra (f_1_(z_1_)) for single cells in suspension from 8 different source-target geometries. Overall, the resultant spectra showed good agreement with the MC generated values. Most notably, the generated single-event spectra qualitatively and quantitatively demonstrate the strength of this approach. In particular, comparing the results across the various source-target spectra indicate the network was able to learn from the training set and predict the output spectra. **Figure 5** shows that the network is equally capable of learning to produce simplistic spectra (i.e., sources located in the nucleus, cytoplasm, and cell surface) as well are more complex spectra (i.e., source located outside the cell). The consistency in accuracy going from simple to more complex spectra serves as a proof of principle for the success of the NN.

In addition to calculating the single-event spectra, we also calculated <z_1_> and <z_1_^2^> values. The use of these moments can simplify calculations, such as predicting cell survival given an inherent cell sensitivity, z_o_ (20). The ranges of errors in the <z_1_> and <z_1_^2^> values proved consistent with previously published values for deviations between MC and analytically tabulated values (27). In the prior study, these differences ranged from roughly −2% to 3% for <z_1_> and −4% to 6% for <z_1_^2^> (27). The errors associated with these values are consistent with those produced using a NN trained specifically on <z_1_> and <z_1_^2^> where the standard deviation in errors for <z_1_> ranged from 1.5-2.1%, while the standard deviation in errors for <z_1_^2^> ranged from 2.6-4.0% (24). Our previous study showed that errors of this magnitude result in an uncertainty in cell survival estimation on the order of +/-3% over a broad range of z_o_ values (24). These errors are well within the uncertainties of cell survival assays.

The data used to train the network was generated primarily through MC data. In theory, the NN is not limited to using MC data, as it can potentially use data from any source, including measured spectral data (33, 34). In this case, the smoothing feature associated with the network may be advantageous as it may reduce some of the inherent experimental error. Moreover, using experimental data allows one to compare the network output to direct measurement, as opposed to comparing against MC-based spectra which may be limited by the assumptions used in the simulation. However, experimental microdosimetric data are often limited, and hence the validation of this proposition is beyond the scope of this study.

An important consideration is that the network is only able to generate single-event spectra for a single geometry at a time. If one were interested in a more complex spectrum, such as those due to a source in the nucleus and cytoplasm, or from multiple alpha particle energies, the network would not be able to produce this directly. Rather, the individual spectra for each source/target configuration and/or energy would need to be generated independently. The composite single-event spectrum would be determined by integrating the individual spectra weighted by the number of alpha particles emitted over the sub-volume (13). Alternatively, if only the individual moments would be required (such as to estimate mean dose and variance in specific energy, or cell survival), these can be calculated from the individual spectra and combined based on the average number of hits from each component (20).

A notable limitation of the current neural network is its restriction to generating single-event specific energy spectra only for the geometries it was explicitly trained on. This limitation arises as the network’s ability to predict spectra is inherently dependent on the configurations it was trained on. As a result, if we were to task the network with generating spectra for an entirely new geometry which it has not encountered before, the network would likely fail to produce accurate results. This is because it lacks the necessary learned patterns to process and generate outputs for untrained geometries. This limitation, however, highlights an opportunity for future development. By expanding the training dataset to encompass a broader array of geometries, the network could be made more adaptable, allowing it to handle a wider variety of configurations.

## Data Availability

The raw data supporting the conclusions of this article will be made available by the authors, without undue reservation.

## Appendix

The algorithm used for generating single-event specific-energy spectra is based on prior studies (19, 27). The input for the simulation consists of the alpha particle energy, cell (r_c_) and nuclear (r_n_) radii and source-target geometry. Eight source-target geometries are considered with activity confined to the cell nucleus, cytoplasm, cell surface or outside the cell for cells in suspension as well as activity that is uniform everywhere, activity that is uniform everywhere except in the cell nucleus, activity located on the cell surfaces and activity only in the cell cytoplasm for cells packed in planes (**Figure 1**). In each case, the nucleus and cell are assumed to be spherical and concentric.

Based on a random number generation, the radial position of the alpha particle decay is generated depending on the chosen source-target geometry for each particle simulated. Only the radial position is required (which increases calculation efficiency) due to the spherical symmetry. Additionally, since the alpha particle has a finite range, only those emissions within a distance of the alpha particle range plus the nuclear radius need to be considered. Each alpha particle emission is assumed to travel in a straight line consistent with previous studies (6, 27, 35). Additionally, delta rays are neglected as their range is much smaller than the nuclei sizes considered in this study (6). For each alpha particle that is simulated, a random angle is generated. If the source is located in the cell nucleus, then the distance to the cell nucleus surface is calculated. If the emission occurs outside the nucleus (in cytoplasm, cell surface or outside the cell), the maximum angle that the point of emission subtends with the nucleus is calculated as follows:

(A.1)
$$\text{cos}\left(\theta_{\text{max}}\right)=\frac{\sqrt{d_{\text{t}}^{2}-r_{\text{n}}^{2}}}{d_{\text{t}}}$$

where *d*_t_ is the distance from the point of emission to the center of the cell nucleus (24, 27) (**Figure 6**). To determine if the alpha particle emission will hit the nucleus, the product of the unitary vectors defining the alpha particle emission is determined. If this value is < cos(θ_max_), then the alpha particle will hit the nucleus. For cases where the alpha particle will hit the nucleus, two intercepts are calculated corresponding to where the alpha particle enters and exits the nucleus, respectively. The difference between the range of the alpha particle and the distance it travels to where it intercepts the nucleus is defined as the residual range (R_res_). For cases where the range of the alpha particle is not long enough to exit the nucleus, only the distance until the alpha particle stops in the nucleus is considered.

The energy deposited in the nucleus is based on the range/energy relationship for alpha particles in water from ICRU 49 (36) with the curve fit (19, 37) for this relationship given by:

(A.2)
$$\begin{array}{c} E_{res}\left(\text{MeV}\right)=1.34\times 10^{-1}+1.89\times 10^{-1}\times R_{res}-2.15\\ \times 10^{-3}\times {R_{res}}^{2}+1.72\times 10^{-5}\times {R_{res}}^{3}\;-\;5.47\\ \times 10^{-8}\times {R_{res}}^{4}\;\;\;\;\;\;\;R_{res}>10\;\text{µm} \end{array}$$

(A.3)
$$\begin{array}{l} E_{res}\left(\text{MeV}\right)=3.51\times 10^{-2}\times R_{res}+3.42\times 10^{-2}\times {R_{res}}^{2}-2.00\\ \times 10^{-3}\times {R_{res}}^{3}\;\;\;\;\;\;\;\;R_{res}\leq 10\;\text{µm} \end{array}$$

In these equations, R_res_ represents the residual range of the alpha particle which is the range minus the distance traversed by the particle. E_res_ represents the amount of energy of an alpha particle with range R_res_. Specific energy (z) is determined by dividing the energy deposited by the mass of the cell nucleus. This curve fit was also compared with data from the more recent ICRU 90 report (38). As shown in **Figure 7**, the curve fit agrees well with these data (R^2^ = 0.999).

Simulations for geometries a-d (**Figure 1**) were performed using an Excel spreadsheet (Microsoft, Redmond, WA, USA) (27). Each simulation used 500,000 particles having a standard error of < 0.2% and was computed in < 3 sec on a standard desktop computer with Intel® Core™ iS-10500 CPU @ 3.10 GHz, 3096 MHz, 6 cores and 12 logical processors. For geometries e-h (**Figure 1**), the same computer was used with code that simulated emissions for the geometry described (19, 37). For these cases, the computational times ranged from 60-90 seconds for a simulation with 100,000,000 particles. Of note, the MC spreadsheet simulated significantly fewer particles since all of them intersected the cell nucleus, while the code used for geometries e-h used more particles since the majority of them did not intersect the cell nucleus.
